# Study of Ferroptosis Transmission by Small Extracellular Vesicles in Epithelial Ovarian Cancer Cells

**DOI:** 10.3390/antiox12010183

**Published:** 2023-01-12

**Authors:** Carmen Alarcón-Veleiro, Rocío Mato-Basalo, Sergio Lucio-Gallego, Andrea Vidal-Pampín, María Quindós-Varela, Thamer Al-Qatarneh, Germán Berrecoso, Ángel Vizoso-Vázquez, María C. Arufe, Juan Fafián-Labora

**Affiliations:** 1 Grupo de Investigación en Terapia Celular y Medicina Regenerativa, Departamento de Fisioterapia, Medicina y Ciencias Biomédicas, Facultad de Ciencias de la Salud, Universidade da Coruña, 15008 A Coruña, Spain; 2 INIBIC-Complejo Hospitalario Universitario A Coruña (CHUAC), Centro Interdisciplinar de Química de Química y Biología (CICA), Universidade da Coruña (UDC),15008 A Coruña, Spain; 3 Translational Cancer Research Group, A Coruña Biomedical Research Institute (INIBIC), Carretera del Pasaje s/n, Complexo Hospitalario Universitario de A Coruña (CHUAC), 15006 A Coruña, Spain; 4 EXPRELA Group, Centro Interdisciplinar de Química de Química y Biología (CICA), Universidade da Coruña (UDC), 15008 A Coruña, Spain; 5 Instituto de Investigación Biomédica de A Coruña (INIBIC), 15006 A Coruña, Spain; 6 Department of Biology, Faculty of Sciences, Campus de A Zapateira, Universidade da Coruña (UDC), 15008 A Coruña, Spain; 7 Center for Research in Molecular Medicine and Chronic Diseases (CiMUS), 15782 Santiago de Compostela, Spain; 8 Instituto de Investigación Sanitaria de Santiago de Compostela (IDIS), IDIS Research Institute, 15706 Santiago de Compostela, Spain; 9 Department of Pharmacy and Pharmaceutical Technology, School of Pharmacy, Universidade de Santiago de Compostela, 15782 Santiago de Compostela, Spain

**Keywords:** ferroptosis, small extracellular vesicles, epithelial ovarian cancer

## Abstract

Epithelial ovarian cancer (EOC) is the most lethal gynecological cancer. The current treatment for EOC involves surgical debulking of the tumors followed by a combination of chemotherapy. While most patients achieve complete remission, many EOCs will recur and develop chemo-resistance. The cancer cells can adapt to several stress stimuli, becoming resistant. Because of this, new ways to fight resistant cells during the disease are being studied. However, the clinical outcomes remain unsatisfactory. Recently, ferroptosis, a novel form of regulated cell death trigged by the accumulation of iron and toxic species of lipid metabolism in cells, has emerged as a promising anti-tumor strategy for EOC treatment. This process has a high potential to become a complementary treatment to the current anti-tumor strategies to eliminate resistant cells and to avoid relapse. Cancer cells, like other cells in the body, release small extracellular vesicles (sEV) that allow the transport of substances from the cells themselves to communicate with their environment. To achieve this, we analyzed the capacity of epithelial ovarian cancer cells (OVCA), treated with ferroptosis inducers, to generate sEV, assessing their size and number, and study the transmission of ferroptosis by sEV. Our results reveal that OVCA cells treated with ferroptotic inducers can modify intercellular communication by sEV, inducing cell death in recipient cells. Furthermore, these receptor cells are able to generate a greater amount of sEV, contributing to a much higher ferroptosis paracrine transmission. Thus, we discovered the importance of the sEV in the communication between cells in OVCA, focusing on the ferroptosis process. These findings could be the beginning form to study the molecular mechanism ferroptosis transmission through sEV.

## 1. Introduction

More than 85% of ovarian cancer has an epithelial origin. It arises from a layer of cells covering the surface of the ovaries or fallopian tubes and accounts for approximately 90% of the primary ovarian tumors. It is the most lethal gynecologic cancer due to the difficult diagnosis derived from the vague and non-specific symptoms at the initial stages [1]. There are some treatments for this cancer, such as angiogenesis inhibitors and Poly (ADP-ribose) polymerases (PARPs) inhibitors, among others. However, the clinical outcomes of these treatments for epithelial ovarian cancer (EOC) are still unsatisfactory [2]. Ferroptosis is a form of regulated cell death that occurs by iron-dependent lipid peroxidation. The ferroptotic cells are characterized by an increase of iron (II) levels, lipid peroxidation products as malondialdehyde (MDA) and a decrease of the ratio reduced glutathione/oxidated glutathione (GSH/GSSG) [3]. It is a promising strategy for inducing the death of cancer cells that are resistant to other therapies. In the last year, it was reported that ferroptosis suppressor protein 1 (FSP1) and the canonical glutathione-dependent GPx4 pathway are important to predict the efficacy of ferroptosis-inducing drugs (erastin and RSL3) in cancer [4,5] because these targets are associated with resistance to ferroptosis in epithelial ovarian cancer cells (OVCA).

The tumor microenvironment (TME) consists of tumor cells, tumor stromal cells including fibroblasts, endothelial, immune cells such as microglia, macrophages and lymphocytes, and the non-cellular components of the extracellular matrix, extracellular vesicles, among others. It is the center of the function of cellular and non-cellular components through the signaling networking to use the non-malignant cells to promote tumorigenesis in all phases of cancer development and metastasis [6].

Small extracellular vesicles (sEV) are nanovesicles of endosomal origin, enriched in nucleic acids, lipids and proteins that mediate intercellular communication at physiological and pathological levels [7]. The tumor-derived sEV proteins are involved in tumor progression, immune regulation and metastasis and they serve as biomarkers for early-stage cancer detection and classify uncertain primary tumor types such as this tumor type [8]. It was reported that the removal of intracellular iron by extracellular vesicles (specifically, by sEV) is an important mechanism driving ferroptosis and increasing the sEV secretion [9,10]. Moreover, sEVs have the capacity to produce changes in the metabolic state of the cells by the regulation of glutathione metabolism and the lipid peroxidation [11]. Because of that, the intercellular communication through sEV is an attractive field to fight OVCA.

## 2. Materials and Methods

### 2.1. Cell Culture

SKVO3 and A2780 cell lines were used as epithelial ovarian cancer cells (OVCA1 and OVCA2, respectively). OVCA1 is a cell line with epithelial morphology from ovarian adenocarcinoma and was obtained from American Type Culture Collection (ATCC, Rockville, MA, USA) and OVCA2 is a cell line from ovarian carcinoma, and it was donated from Dr. Vizoso-Vázquez (EXPRELA Group, UDC, A Coruña, Spain).

Human umbilical cords (UC) were obtained from caesarean sections performed on healthy women at the Maternity Facility at Complejo Hospitalario Universitario A Coruña (CHUAC). All tissues were obtained with fully informed consent and ethical approval by the supervisor of the Ethical Committee (CEIC: 2019/026) of Galicia. All the women were between 26 and 35 years of age. Mesenchymal stem cells (MSCs) were isolated from UC using the protocol developed by Arufe’s group [12]. Briefly, the tissue was washed with phosphate-buffered saline and cut into small pieces (explants). These explants were then incubated for three five-minute periods in an enzyme mixture containing 1.2-U/mL dispase and 112-U/mL type I collagenase (all from Sigma-Aldrich, Madrid, Spain) and cultured in Dulbecco’s Modified Eagle’s Medium with 10% (v/v) fetal bovine serum, 1% (v/v) penicillin and 1% (v/v) streptomycin (all from Sigma-Aldrich, Madrid, Spain) and growth adhered to the plastic plate. After three days, the explants were removed from the plate, leaving the attached UC-MSCs, which were then cultured in a monolayer in the same medium. When the cells were in passage 4 and 90% confluent, they were removed from the plate using 2% (v/v) trypsin (Sigma-Aldrich, Madrid, Spain) in phosphate-buffered saline to seed in the plates to perform the experiments.

HFFF2 (Human Caucasian fetal foreskin fibroblasts) were donated by Dr. Díaz-Prado (UDC, SP). Cells were maintained in high-glucose, pyruvate, Dulbecco’s modified Eagle’s medium with 10% fetal bovine serum and 1% (v/v) antibiotic-antimycotic solution in a 37 °C incubator with 5% CO_2_.

### 2.2. Treatment of Ferroptosis Modulators

OVCA were seeded at a 50% confluence. The next day, the modulation of ferroptosis was started. Three days later, the drugs at suitable concentrations were refreshed up to six days (Table 1).

### 2.3. Isolation of CM, SN and sEVs

The cells were cultured with DMEM supplemented with 10% (v/v) sEV-depleted FBS and 1% (v/v) penicillin and 1% (v/v) streptomycin (all from Life Technologies, Carlsbad, CA, USA). Cells were cultured to 80% confluence, and the supernatants were collected after 72 h post-treatment. For the dissection of conditioned medium (CM), whole CM (10 mL/10 cm dish) was collected and centrifuged at 2000× *g* for 10 min to eliminate cell debris. One half (5 mL) was used as whole CM, concentrated, and used to treat cells, and the other half (5 mL) was further processed.

The CM was further processed using a sterile 0.22-µm filter (GE Healthcare Life Sciences, Little Chalfont, UK) and they were transferred into new ultracentrifugation tubes (Hitachi, Chiyoda, Tokyo, Japan) and centrifuged at 100,000× *g* for 2 h at 4 °C in a Hitachi CP100NX (Hitachi, Chiyoda, Tokyo, Japan) ultracentrifuge with a P70AT rotor (Hitachi, Chiyoda, Tokyo, Japan).

The last supernatants containing sEV-depleted FBS were collected as denominated supernatant fraction (SN), and the pellets (sEV fraction) were resuspended at 200 µL PBS (MP Biomedicals, Illkrich-Graffenstaden, France) or medium depending on if they were used for characterization or functional assays.

All relevant data of our experiments were submitted to the EV-TRACK knowledgebase (EV-TRACK ID: EV220413) [13].

### 2.4. Treatment with CM, SN and sEV

A total of 5 mL of CM and SN were concentrated using a 10K column (Amicon Ultra-0.5 Filter) at 14,000 g for 10 min, obtaining a concentration factor 10x. Later, they were re-suspended in 1.2 mL DMEM 10% (v/v) FBS 1% (v/v) P/S. 100 µL/well were used to treat 12 wells of a 96-well plate. The OVCA cells were plated and one day later they were treated with sEV from ferroptotic cells at day 0 and 3 for six days with the same number (2 × 10^6^ particles) of sEV in DMEM 10% (v/v) FBS-depleted sEV and 1% (v/v) penicillin and 1% (v/v) streptomycin (all from Sigma-Aldrich, Madrid, Spain).

### 2.5. Viability Assay

The number of cells was evaluated by the colorimetric MTT assay (Cell Proliferation Kit I, Roche, Basel, Switzerland) according to manufacturer’s instructions. The absorbance was measured with a NanoQuant microplate reader (Tecan Trading AG, Männedorf, Switzerland) at 540 nm.

### 2.6. Crystal Violet Staining

The plates were fixed using 4% (v/v) paraformaldehyde for 15 min at room temperature and washed three times with PBS. Then, the plates were stained with 0.5% (v/v) crystal violet solution and scanned. The quantification of crystal violet was performed by solubilizing crystal violet staining (Sigma-Aldrich, Madrid, Spain) with 30% (v/v) acetic acid and measuring the absorbance at 590 nm using a NanoQuant microplate reader (Tecan Trading AG, Männedorf, Switzerland).

### 2.7. Lipid Peroxidation Assay

The levels of malondialdehyde (MDA) in cells were determined using the Lipid peroxidation assay colorimetric following the manufacturer’s instructions (Canvax, Córdoba, Spain). The absorbance was measured at 532 nm using a NanoQuant microplate reader (Tecan Trading AG, Männedorf, Switzerland).

### 2.8. Detection of GSH/GSSG

Oxidized glutathione (GSSG) was measured as follows; briefly, after Pyroglutamic acid extraction (Sigma-Aldrich, Madrid, Spain), reduced glutathione (GSH) was derivatized with 2-vinylpyridine at room temperature for 1 h, and the reaction was monitored as follows. Reduced GSH was obtained by subtracting GSSG levels from total glutathione levels. To determine the ratio GSH:GSSG in cells and serum a luminescent-based assay kit (Promega, WI, USA) was used.

### 2.9. Nanoparticle Tracking Analysis (NTA)

The NanoSight® NS300 (Malvern Instruments, Malvern, UK) was calibrated using Silica Microspheres beads. sEV were diluted in PBS to obtain a particle concentration between 10^7^ and 10^8^ particles/mL. Three measurements of 60 s were taken per each sample and the mean value was used to determine particle number. The movement of each particle in the field of view was measured to generate the average displacement of each particle per unit time, which was calculated using the NTA 3.0 software.

### 2.10. Detection of ROS

Reactive oxygen species (ROS) in cells were estimated as following; cell pellet was homogenized in ice-cold Tris-HCl buffer (40 mM, pH 7.4). Samples were mixed with 2′, 7′-dichlorofluorescein diacetate (1 μM) prepared in Tris-HCl buffer (40 mM, pH 7.4). The mixture was incubated for 30 min at 37 °C. Finally, the fluorescence intensity of the samples was measured using a NanoQuant microplate reader (Tecan Trading AG, Männedorf, Switzerland) (λ excitation 485 nm and λ emission 525 nm).

### 2.11. Determination Intracellular Iron (II) Levels

Iron Assay Kit (ThermoFisher Scientific) was used to determine the amount of iron (II) present in cell samples and sEV as a marker for ferroptosis. Absorbance at 592 nm was measured using a NanoQuant microplate reader (Tecan Trading AG, Männedorf, Switzerland).

### 2.12. Pre-Treatment of sEV with Triton

A total of 2 × 10^6^ particles sEV were treated with 0.075% (w/w) Triton X-100 (Sigma-Aldrich, Madrid, Spain), vortexed 30 s at room temperature. Then, the sEV were washed with 5 mL PBS and ultracentrifuged at 100,000× *g* for 2 h at 4 °C in an Optimal-90K ultracentrifuge with a 60 Ti rotor (Beckman Coulter, Mississauga, ON, Canada).

### 2.13. Immunodetection by WB

The proteins of sEV were isolated using the RIPA lysis buffer (150 mM sodium chloride, 50 mM Tris-base, 0.1% (p/v) sodium dodecyl sulfate, triton 1% (v/v) (all from Sigma-Aldrich, San Diego, CA, USA)). Lysates were diluted in 4X laemmli buffer (BioRad, Hercules, CA, USA) obtaining the same concentration of proteins for each sample. The proteins were separated in SDS-PAGE gels and transferred into PVDF membrane with 0.45 mm pore size (Millipore, Burlington, MA, USA). This membrane incubates for 1 h with the Blocking buffer (5% bovine serum albumin) (Sigma-Aldrich, San Diego, CA, USA). Then, we add the primary antibody at suitable concentration (Table 2), which is incubated overnight at 4 °C. The following day the membrane is washed at least 3 times 10 min before adding the secondary antibody which will be 1 h incubating. Another 3 washes are made before revealing the protein expression with the ECL substrate (Millipore, MA, USA).

### 2.14. RNA Isolation, Synthesis cDNA and qPCR-RT

Total RNA was extracted using TRIzol Reagent (Thermo Fisher, Foster City, CA, USA) according to the manufacturer’s instructions. cDNA synthesis was performed using a High-Capacity cDNA Reverse Transcriptase kit (Thermo Fisher, Foster City, CA, USA). qPCR reactions were performed using SYBR Green PCR Master Mix (Applied Biosystems, Foster City, CA, USA) and the primers in Table 3 in a LightCycler 480 (Roche, Basel, Switzerland).

### 2.15. Statistics

Data analysis and graphs were conducted via GraphPad Prism 8.0 statistical software (GraphPad Software Inc., San Diego, CA, USA). The results are expressed as the mean ± standard deviation. We will compare the means of the variables studied between the different treatments after checking normality with the Kolmogorov–Smirnov test, with the *t* test for paired samples, or the Wilcoxon test, according to procedure. A value of * *p* < 0.05 and ** *p* < 0.01 was indicative of a significant difference.

## 3. Results

### 3.1. Erastin and RSL3 Induce Ferroptosis in Ovarian Epithelial Cancer Cells (OVCA)

**Figure 1:**
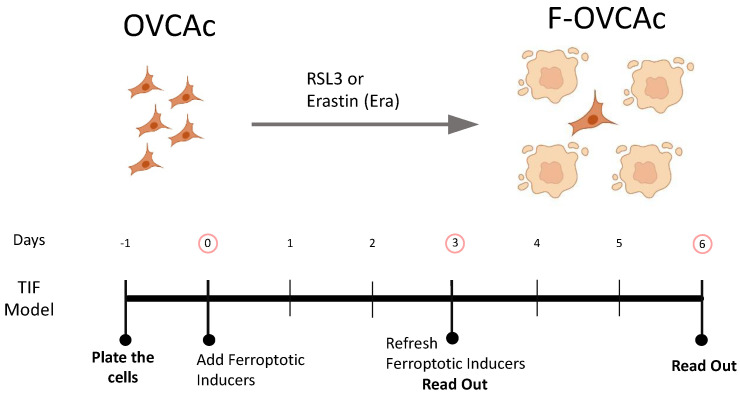
Schematic representation of ferroptosis induction in OVCAc. OVCAc: epithelial ovarian cancer cells; TIF: Therapy-induced ferroptosis; F-OVCAc: Ferroptotic Epithelial ovarian cancer cells.

**Figure 2:**
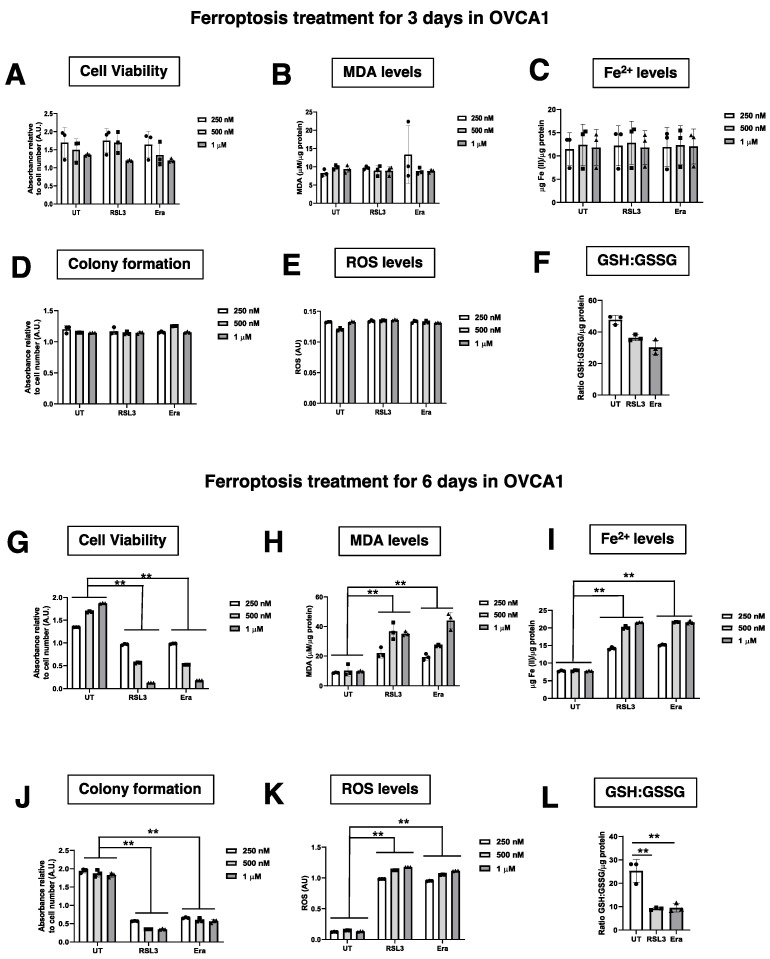
Evaluation of ferroptosis induction in OVCAc. (**A**) Histogram of cell viability in OVCAc treated with several concentrations (250 nM, 500 nM and 1 µM) of ferroptotic inducers ((1S,3R)-RSL3 (RSL3) and Erastin (Era)) for 3 days measured by MTT assay in OVCA1; (**B**) Histogram of Malondialdehyde (MDA) levels in OVCAc treated with several concentrations (250 nM, 500 nM and 1 µM) of ferroptotic inducers ((1S,3R)-RSL3 (RSL3) and Erastin (Era)) for 3 days in OVCA1; (**C**) Histogram of Iron (II) (Fe^2+^) levels in OVCAc treated with several concentrations (250 nM, 500 nM and 1 µM) of ferroptotic inducers ((1S,3R)-RSL3 (RSL3) and Erastin (Era)) for 3 days; (**D**) Histogram of colony formation in OVCAc treated with several concentrations (250 nM, 500 nM and 1 µM) of ferroptotic inducers ((1S,3R)-RSL3 (RSL3) and Erastin (Era)) for 3 days in OVCA1; (**E**) Histogram of reactive oxygen species (ROS) levels in OVCAc treated with several concentrations (250 nM, 500 nM and 1 µM) of ferroptotic inducers ((1S,3R)-RSL3 (RSL3) and Erastin (Era)) for 3 days in OVCA1; (**F**) Histogram of ratio GSH:GSSG in OVCAc treated with 500 nM of ferroptotic inducers ((1S,3R)-RSL3 (RSL3) and Erastin (Era)) for 3 days in OVCA1; (**G**) Histogram of cell viability in OVCAc treated with several concentrations (250 nM, 500 nM and 1 µM) of ferroptotic inducers ((1S,3R)-RSL3 (RSL3) and Erastin (Era)) for 6 days measured by MTT assay in OVCA1; (**H**) Histogram of Malondialdehyde (MDA) levels in OVCAc treated with several concentrations (250 nM, 500 nM and 1 µM) of ferroptotic inducers ((1S,3R)-RSL3 (RSL3) and Erastin (Era)) for 6 days in OVCA1; (**I**) Histogram of Iron (II) (Fe^2+^) levels in OVCAc treated with several concentrations (250 nM, 500 nM and 1 µM) of ferroptotic inducers ((1S,3R)-RSL3 (RSL3) and Erastin (Era)) for 6 days in OVCA1; (**J**) Histogram of colony formation in OVCAc treated with several concentrations (250 nM, 500 nM and 1 µM) of ferroptotic inducers ((1S,3R)-RSL3 (RSL3) and Erastin (Era)) for 6 days in OVCA1; (**K**) Histogram of reactive oxygen species (ROS) levels in OVCAc treated with several concentrations (250 nM, 500 nM and 1 µM) of ferroptotic inducers ((1S,3R)-RSL3 (RSL3) and Erastin (Era)) for 6 days in OVCA1; (**L**) Histogram of ratio GSH:GSSG in OVCAc treated with 500 nM of ferroptotic inducers ((1S,3R)-RSL3 (RSL3) and Erastin (Era)) for 6 days in OVCA1. The graphs show the mean ± SD of three independent experiments. ** *p* < 0.01.

**Figure A1:**
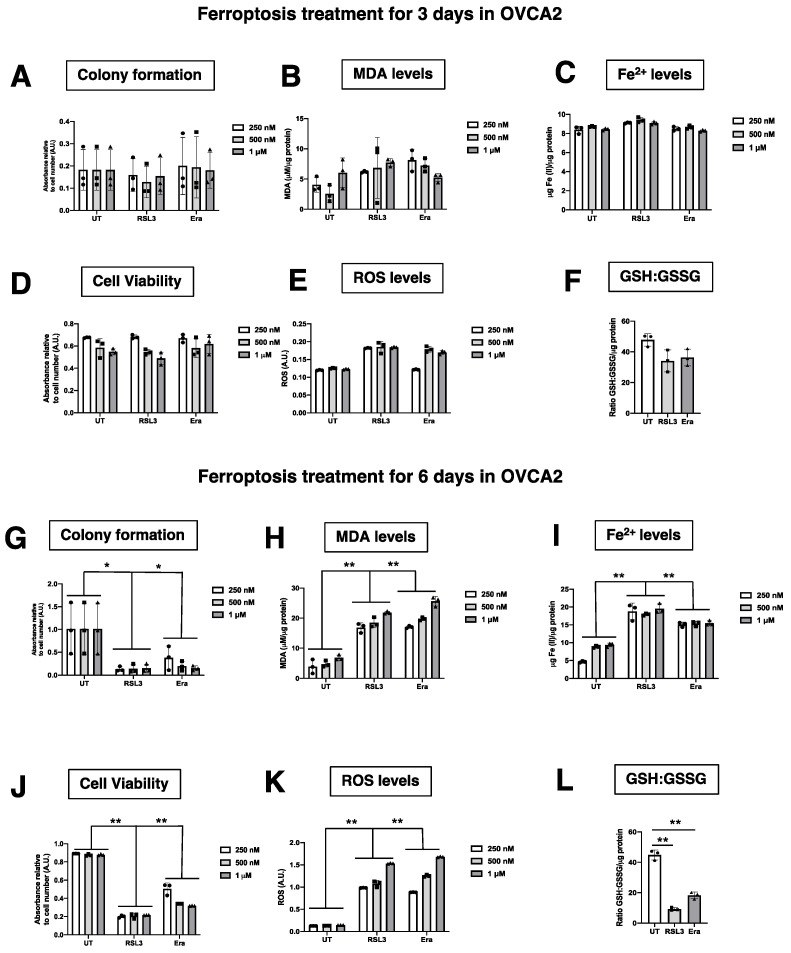
Analysis of ferroptosis in F-OVCAc. (**A**) Histogram of colony formation in OVCAc treated with several concentrations (250 nM, 500 nM and 1 µM) of ferroptotic inducers ((1S,3R)-RSL3 (RSL3) and Erastin (Era)) for 3 days measured by MTT assay in OVCA2; (**B**) Histogram of Malondialdehyde (MDA) levels in OVCAc treated with several concentrations (250 nM, 500 nM and 1 µM) of ferroptotic inducers ((1S,3R)-RSL3 (RSL3) and Erastin (Era)) for 3 days in OVCA2; (**C**) Histogram of Iron (II) (Fe^2+^) levels in OVCAc treated with several concentrations (250 nM, 500 nM and 1 µM) of ferroptotic inducers ((1S,3R)-RSL3 (RSL3) and Erastin (Era)) for 3 days in OVCA2; (**D**) Histogram of cell viability in OVCAc treated with several concentrations (250 nM, 500 nM and 1 µM) of ferroptotic inducers ((1S,3R)-RSL3 (RSL3) and Erastin (Era)) for 3 days measured by MTT assay in OVCA2; (**E**) Histogram of reactive oxygen species (ROS) levels in OVCAc treated with several concentrations (250 nM, 500 nM and 1 µM) of ferroptotic inducers ((1S,3R)-RSL3 (RSL3) and Erastin (Era)) for 3 days in OVCA2; (**F**) Histogram of ratio GSH:GSSG in OVCAc treated with 500 nM of ferroptotic inducers ((1S,3R)-RSL3 (RSL3) and Erastin (Era)) for 3 days in OVCA2; (**G**) Histogram of colony formation in OVCAc treated with several concentrations (250 nM, 500 nM and 1 µM) of ferroptotic inducers ((1S,3R)-RSL3 (RSL3) and Erastin (Era)) for 3 days in OVCA2; (**H**) Histogram of Malondialdehyde (MDA) levels in OVCAc treated with several concentrations (250 nM, 500 nM and 1 µM) of ferroptotic inducers ((1S,3R)-RSL3 (RSL3) and Erastin (Era)) for 3 days in OVCA2; (**I**) Histogram of Iron (II) (Fe^2+^) levels in OVCAc treated with several concentrations (250 nM, 500 nM and 1 µM) of ferroptotic inducers ((1S,3R)-RSL3 (RSL3) and Erastin (Era)) for 3 days in OVCA2; (**J**) Histogram of cell viability in OVCAc treated with several concentrations (250 nM, 500 nM and 1µM) of ferroptotic inducers ((1S,3R)-RSL3 (RSL3) and Erastin (Era)) for 6 days measured by MTT assay in OVCA2; (**K**) Histogram of reactive oxygen species (ROS) levels in OVCAc treated with several concentrations (250 nM, 500 nM and 1 µM) of ferroptotic inducers ((1S,3R)-RSL3 (RSL3) and Erastin (Era)) for 3 days in OVCA2; (**L**) Histogram of ratio GSH:GSSG in OVCAc treated with 500 nM of ferroptotic inducers ((1S,3R)-RSL3 (RSL3) and Erastin (Era)) for 6 days in OVCA2. The graphs show the mean ± SD of three independent experiments * *p* < 0.05 and ** *p* < 0.01. Related to Figure 2.

To investigate the paracrine ferroptosis in two OVCA cell lines (OVCA1 and OVCA2), two models of ferroptosis were established: (1) RSL3-induced ferroptosis (RSL3): OVCA cells were treated with RSL3, which inhibits the activity of glutathione peroxidase 4 (GPX4) [5], at three concentrations (250 nM, 500 nM and 1 µM) for 3 or 6 days; and (2) Erastin-induced ferroptosis (Era): OVCA cells were treated with Erastin, that induces ferroptosis through the inhibition of the cystine-glutamate transport receptor (systems Xc^-^), the voltage-dependent anion channel (VDAC) and p53 [10], at three concentrations (250 nM, 500 nM and 1 µM) for 3 or 6 days (Figure 1). DMSO treatment was used in control groups (UT). After the treatments, cellular growth capacity was measured by colony formation assay and cell viability. There were not statistically significant differences between OVCA UT cells compared to OVCA cells treated with RSL3 and Erastin at increasing concentrations (250 nM, 500 nM and 1 µM) for 3 days (Figure 2D and Figure A1A). However, a statistically significant decrease in the colony formation and cell viability of the cells treated with RSL3 and Erastin was observed compared to UT at three concentrations after six treatment days (Figure 2J and Figure A1E). Thus, ferroptosis parameters such as concentration of lipid peroxidation product (MDA) and intracellular Fe^2+^ levels were measured in OVCA cells treated with RSL3 and Era to confirm the ferroptosis stage in the cells after three (Figure 2B,C and Figure A1B,C) and six days of treatments (Figure 2H,I and Figure A1F,G). The levels of MDA and intracellular Fe^2+^ were not increased in OVCA cells treated with RSL3 and Era for three days (Figure 2B,C and Figure A1B,C). Moreover, OVCA treated with RSL3 and Era had a statistically significant increase in MDA and Fe^2+^ levels after six days of treatment. (Figure 2H,I and Figure A1F,G). Moreover, it was confirmed that the ferroptosis induction with the ratio of GSH/GSSG in the OVCA cells treated with RSL3 and Era compared to UT tended to decrease at three days of treatment (Figure 2F and Figure A1F). However, until three days later, there were statistically significant lower levels of GSH/GSSG in RSL3 and Era compared to UT (Figure 2L and Figure A1L). To confirm the therapy-induced ferroptosis (TIF) in OVCA cells using ferroptotic inducers such as RSL3 and Era, oxidative stress directly associated with high intracellular Fe^2+^ levels was evaluated by the analysis of intracellular reactive oxygen species (ROS) (Figure 2E,K and Figure A1D,H) and the cell viability using MTT assay (Figure 2A,G and Figure A1D,J). ROS levels were statistically higher in OVCA treated with RSL3 and Era in comparison to UT after the treatment for six days (Figure 2K and Figure A1H). Thus, the cell viability was decreased statistically significant with six days of treatment (Figure 2G and Figure A1J). These data show that the pharmacological inhibitors (RSL3 and Erastin) induce ferroptosis in OVCA cells and could be used as an attractive strategy based on Therapy-induced Ferroptosis (TIF).

### 3.2. TIF in OVCA Modulates the Production of sEV in Ferroptotic OVCA (F-OVCA)

**Figure 3:**
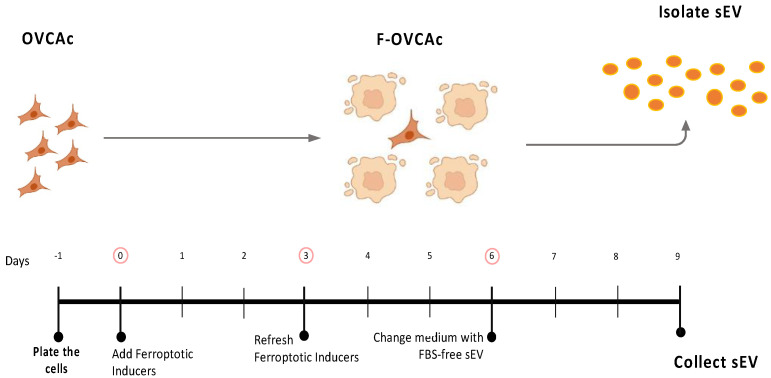
Schematic representation of isolation of small extracellular vesicles (sEV) from ferroptotic epithelial ovarian cancer cells (F-OVCAc).

**Figure A2:**
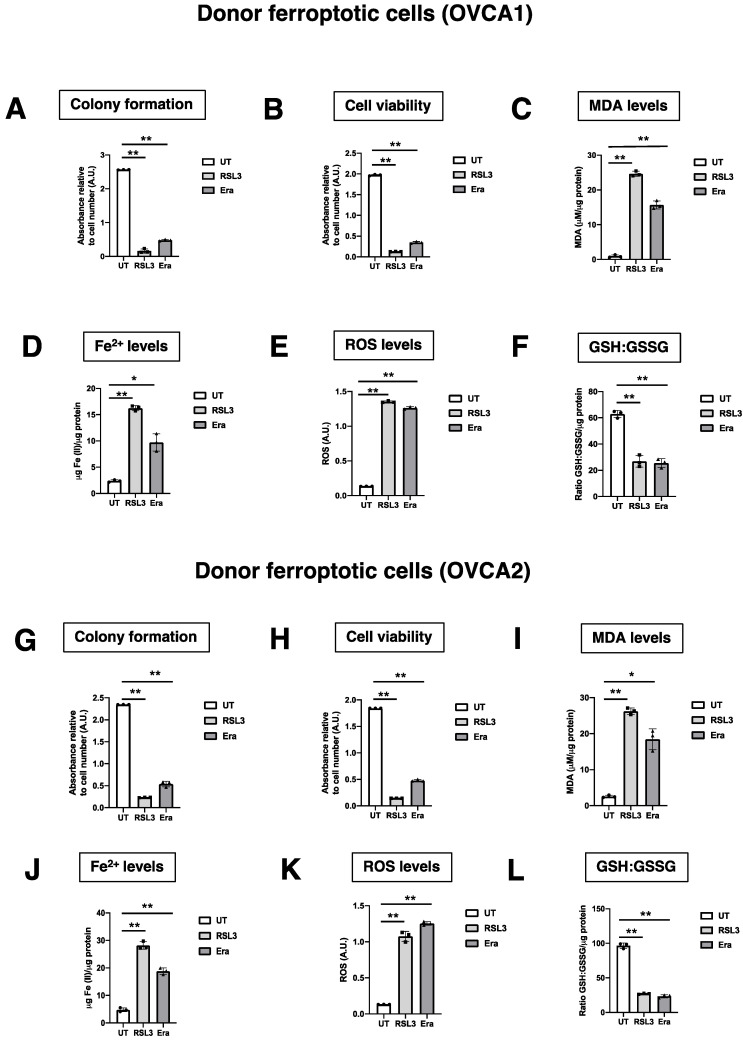
Ferroptosis parameters in donor ferroptotic cells used for secondary ferroptosis paracrine. (**A**) Histogram of colony formation in F-OVCAc (OVCA1) at day 9; (**B**) Histogram of cell viability using MTT assay in F-OVCAc (OVCA1) at day 9 in OVCA1; (**C**) Histogram of Malondialdehyde (MDA) levels in F-OVCAc (OVCA1) at day 9; (**D**) Histogram of Iron (II) (Fe^2+^) levels in F-OVCAc (OVCA1) at day 9; (**E**) Histogram of reactive oxygen species (ROS) levels in OVCAc (OVCA1) at day 9; (**F**) Histogram of ratio GSH:GSSG in F-OVCAc (OVCA1) at day 9; (**G**) Histogram of colony formation in F-OVCAc (OVCA2) at day 9; (**H**) Histogram of cell viability using MTT assay in F-OVCAc (OVCA2) at day 9 in OVCA1; (**I**) Histogram of Malondialdehyde (MDA) levels in F-OVCAc (OVCA2) at day 9; (**J**) Histogram of Iron (II) (Fe^2+^) levels in F-OVCAc (OVCA2) at day 9; (**K**) Histogram of reactive oxygen species (ROS) levels in OVCAc (OVCA2) at day 9; (**L**) Histogram of ratio GSH:GSSG in F-OVCAc (OVCA2) at day 9. The graphs show the mean ± SD of three independent experiments. * *p* < 0.05 and ** *p* < 0.01. Related to Figure 4.

To assess the capacity of the ferroptosis induction to change intercellular communication by sEV, OVCA were treated with RSL3 and Era. After six days, the influence of ferroptosis inducers on OVCA cells was removed by PBS washing and the addition of fresh CM without the drugs (Figure 1). Then, the conditioned medium (CM) was collected after three days and the sEV were isolated by ultracentrifugation following the previous group’s studies [11,14] (Figure 3). The ferroptotic phenotype in the cells was confirmed by colony formation assay (Figure A2A,G), cell viability (Figure A2B,H), levels of MDA (Figure A2C,I), Fe^2+^ (Figure A2D,J), ROS (Figure A2E,K) and ratio GSH:GSSG (Figure A2F,L).

**Figure 4:**
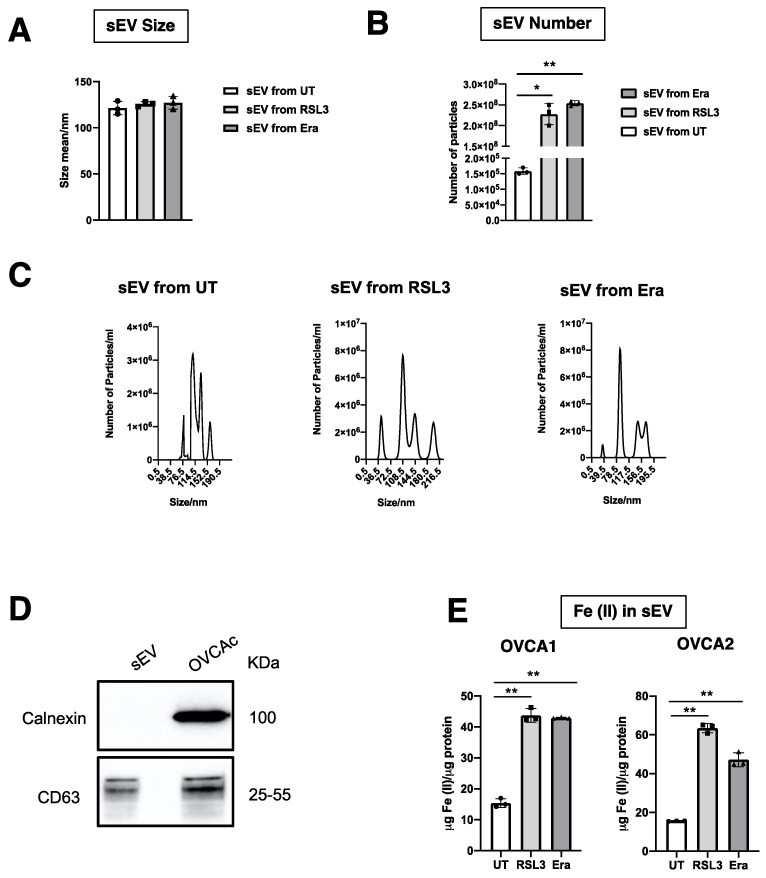
Characterization of sEV from F-OVCAc. (**A**) Mean hydrodynamic diameter size and (**B**) number of sEV from F-OVCAc measured by Nanoparticle Tracking Analysis (NTA) in OVCA1; (**C**) Histogram distribution of sEV from UT, RSL3 and Era by Nanoparticle Tracking Analysis (NTA) in OVCA1; (**D**) EV-related marker (CD63) and lack of contaminant (Calnexin) in cells and sEV by WB in OVCA1; (**E**) Histogram of Iron (II) (Fe^2+^) levels in sEV from OVCAc treated with 500 nM of ferroptotic inducers ((1S,3R)-RSL3 (RSL3) and Erastin (Era)) for 6 days in OVCA1 and OVCA2. The graphs show the mean ± SD of three independent experiments. * *p* < 0.05, ** *p* < 0.01.

The diameter and production of sEV were measured by NTA. It was observed that the diameter of sEV was around 125 nm, similar to the previous group’s results, and there was not statistical significance from UT, RSL3 and Era [11,14,15] (Figure 4A,C). With respect to sEV number, a statistically significant increase in the production of sEV was found in F-OVCA (RSL3 and Era) in comparison with UT (Figure 4B,C). The sEV were characterized by WB to confirm the presence of an sEV marker (CD63) and the absence of endoplasmic reticulum contaminant (Calnexin) (Figure 4D). Additionally, the Fe^2+^ levels in the sEV from ferroptotic cells (RSL3 and Era) were quantified in comparison with non-ferroptotic (UT). Statistically significant increase in the levels of Fe^2+^ in F-OVCA (OVCA1 and OVCA2) (Figure 4E) were observed.

### 3.3. F-OVCA Derived sEV Induce Paracrine Ferroptosis

**Figure 5:**
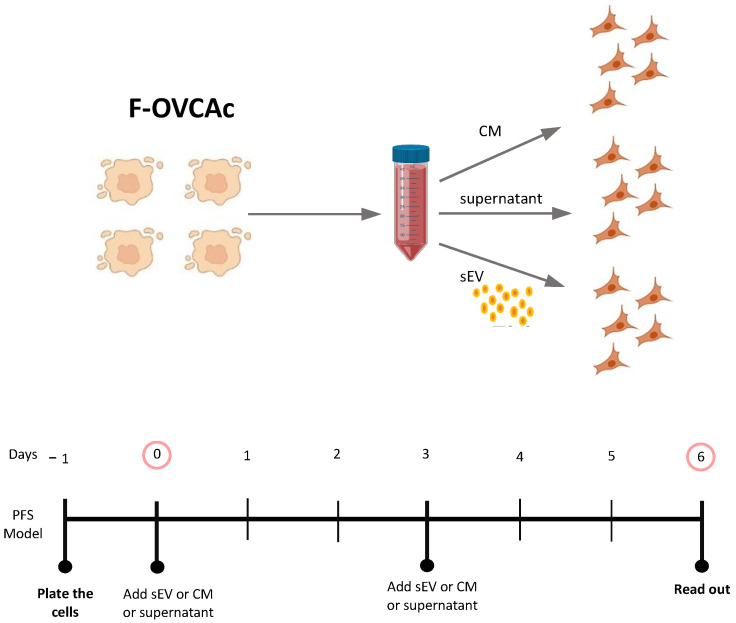
Schematic representation of OVCAc treated with conditioned medium (CM) and two fractions of CM (Supernatant (SN) and sEV) from F-OVCAc.

**Figure 6:**
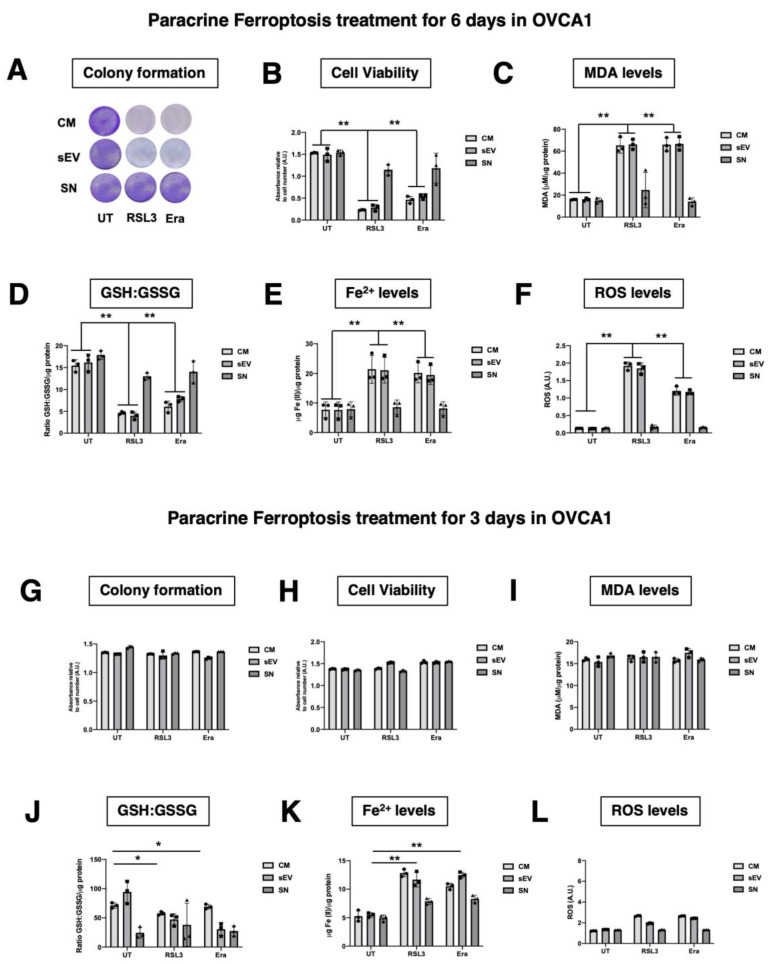
Functionality Analysis of CM and different fractions (SN or sEV) of the conditioned medium of F-OVCAs on OVCAs. (**A**) Representative images of colony formation in OVCAc treated with CM, SN or sEV from F-OVCAc for 6 days in OVCA1; (**B**) Histogram of cell viability by MTT assay in OVCAc treated with SN or sEV from F-OVCAc for 6 days in OVCA1; (**C**) Histogram of Malondialdehyde (MDA) levels in OVCAc treated with CM, SN or sEV from F-OVCAc for 6 days in OVCA1; (**D**) Histogram of ratio GSH/GSSG (Reduced Glutathione/Oxidized Glutathione) levels in OVCAc treated with CM, SN or sEV from F-OVCAc for 6 days in OVCA1; (**E**) Histogram of Iron (II) (Fe^2+^) levels in OVCAc treated with CM, SN or sEV from F-OVCAc for 6 days in OVCA1; (**F**) Histogram of reactive oxygen species (ROS) levels in OVCAc treated with CM, SN or sEV from F-OVCAc for 6 days in OVCA1; (**G**) Histogram of colony formation in OVCAc treated with CM, SN or sEV from F-OVCAc for 3 days in OVCA1; (**H**) Histogram of cell viability by MTT assay in OVCAc treated with CM, SN or sEV from F-OVCAc for 3 days in OVCA1; (**I**) Histogram of Malondialdehyde (MDA) levels in OVCAc treated with CM, SN or sEV from F-OVCAc for 3 days in OVCA1; (**J**) Histogram of ratio GSH/GSSG (Reduced Glutathione/Oxidized Glutathione) levels in OVCAc treated with CM, SN or sEV from F-OVCAc for 3 days in OVCA1; (**K**) Histogram of Iron (II) (Fe^2+^) levels in OVCAc treated with CM, SN or sEV from F-OVCAc for 3 days in OVCA1; (**L**) Histogram of reactive oxygen species (ROS) levels in OVCAc treated with CM, SN or sEV from F-OVCAc for 3 days in OVCA1. The graphs show the mean ± SD of three independent experiments. * *p* < 0.05 and ** *p* < 0.01.

**Figure A3:**
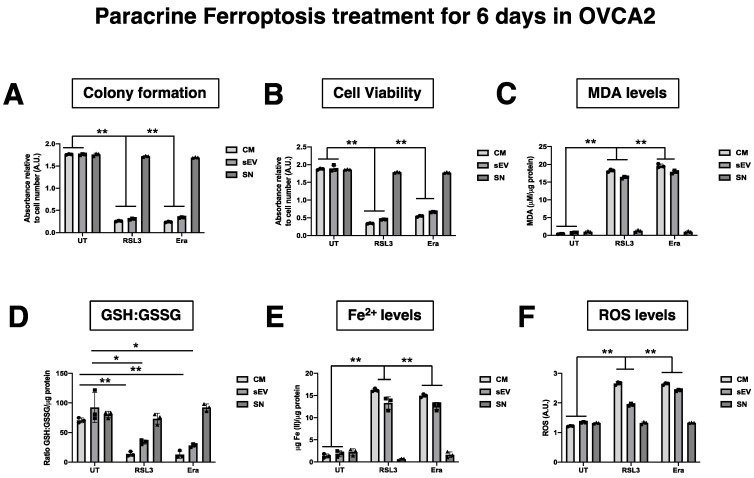
Analysis of ferroptosis paracrine in F-OVCAc treated with CM, sEV and SN for 3 days. (**A**) Histogram of colony formation in OVCAc treated with CM, SN or sEV from F-OVCAc for 6 days in OVCA2; (**B**) Histogram of cell viability by MTT assay in OVCAc treated with CM, SN or sEV from F-OVCAc for 6 days in OVCA2; (**C**) Histogram of Malondialdehyde (MDA) levels in OVCAc treated with CM, SN or sEV from F-OVCAc for 6 days in OVCA2; (**D**) Histogram of ratio GSH/GSSG (Reduced Glutathione/Oxidized Glutathione) levels in OVCAc treated with CM, SN or sEV from F-OVCAc for 6 days in OVCA2; (**E**) Histogram of Iron (II) (Fe^2+^) levels in OVCAc treated with CM, SN or sEV from F-OVCAc for 6 days in OVCA2; (**F**) Histogram of reactive oxygen species (ROS) levels in OVCA treated with CM, SN or sEV from F-OVCAc for 6 days in OVCA2. The graphs show the mean ± SD of three independent experiments. * *p* < 0.05 and ** *p* < 0.01. Related to Figure 6.

To investigate the ferroptosis paracrine transmission in EOC, CM was dissected into supernatant (SN) and sEV fraction (sEV) and the fraction that could be involved was determined. OVCA cells were treated with CM, SN and sEV from F-OVCA, induction previously described (Figure 1), for three and six days (Figure 5) in the cell line OVCA1. After the treatment, evaluation was carried out in the recipient cells: proliferation capacity using colony formation assay (Figure 6A,G), cell viability (Figure 6B,H), ferroptosis signature (levels of MDA, ratio GSH/GSSG, levels Fe^2+^) and intracellular ROS (Figure 6C–F,I–L). SN from F-OVCA (RSL3 and Era) had no effect on recipient cells treated with SN for three or six days. In OVCA treated with F-OVCA derived CM and sEV for six days, a statistically significant decrease in the proliferation capacity, cell viability, and levels of ratio GSH/GSSG (Figure 6D) were observed, and a statistically significant increase in the levels of MDA (Figure 6C), Fe^2+^ (Figure 6E) and intracellular ROS (Figure 6F). The results were validated in the other cell line (OVCA2) (Figure A3). Moreover, CM and, in particular, sEV, can transmit the ferroptosis message in OVCA cells by sEV in paracrine way (Figure 6 and Figure A3).

**Figure 7:**
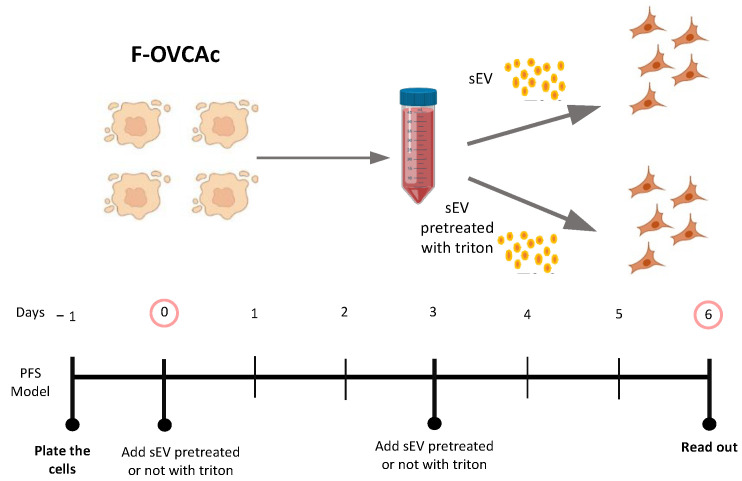
Schematic representation of ferroptotic paracrine transmission using defunctionalized sEV by Triton-X.

**Figure 8:**
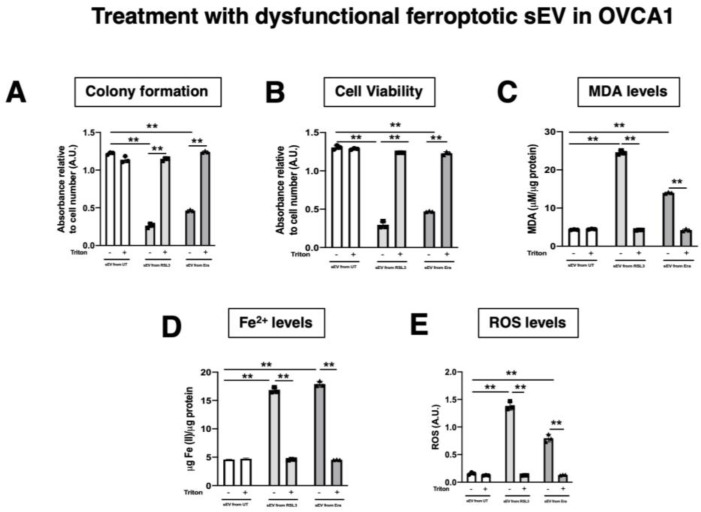
Determination inhibition of ferroptosis paracrine transmission using defunctionalized sEV by Triton-X. (**A**) Histogram of colony formation in OVCAc treated with sEV from F-OVCAc for 6 days using crystal violet assay; (**B**) Histogram of cell viability using MTT assay in OVCAc treated with sEV with/without pre-treatment with Triton-X from F-OVCAc for 6 days in OVCA1; (**C**) Histogram of Malondialdehyde (MDA) levels in OVCAc treated with sEV with/without pre-treatment with Triton-X from F-OVCAc for 6 days in OVCA1; (**D**) Histogram of Iron (II) (Fe^2+^) levels in OVCAc treated with sEV with/without pre-treatment with Triton-X from F-OVCAc for 6 days in OVCA1; (**E**) Histogram of reactive oxygen species (ROS) levels in OVCAc treated sEV with/without pre-treatment with Triton-X from F-OVCAc for 6 days in OVCA1. The graphs show the mean ± SD of three independent experiments. ** *p* < 0.01.

The functionality of sEV was corroborated following the MISEV2018 guidelines [16] using the detergent Triton-X. To defunctionalize, the sEV OVCA cells were treated with the same number of sEV with/without Triton-X pre-treatment for six days (Figure 7). After that, the ferroptotic parameters were evaluated in the recipient cells (OVCA1 and OVCA2). It was observed that sEV pre-treated with Triton-X cannot induce paracrine ferroptosis (Figure A3). Therefore, the capacity of sEV as responsible in the ferroptosis paracrine transmission in EOC was confirmed (Figure 8).

### 3.4. Pharmacological Inhibitors of Ferroptosis Prevent the Ferroptosis Paracrine in OVCA Cells

**Figure 9:**
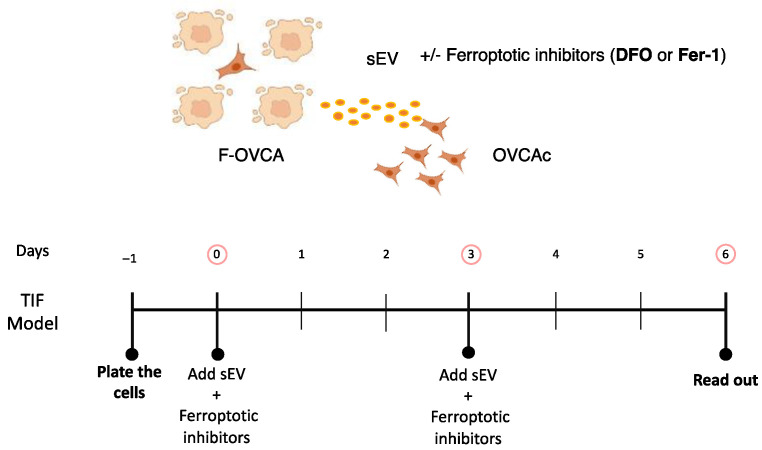
Schematic representation of ferroptotic paracrine through sEV inhibition.

**Figure 10:**
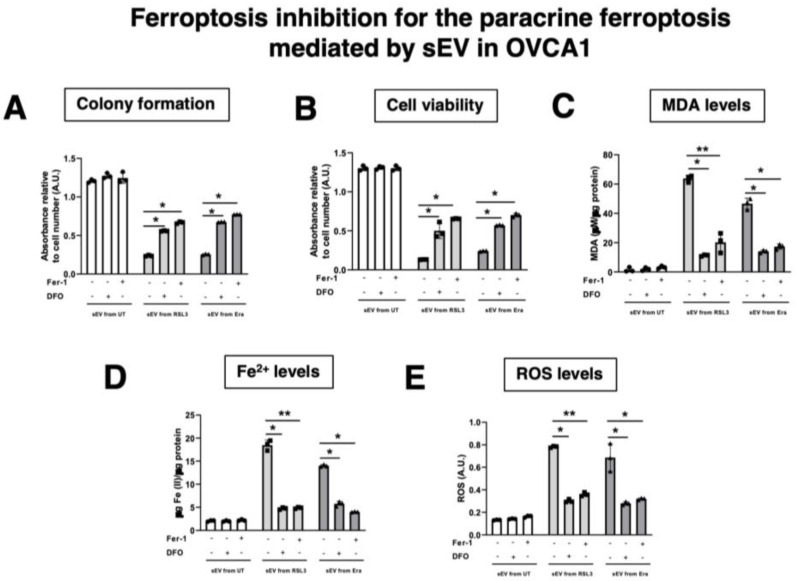
Determination inhibition for ferroptosis paracrine transmission by sEV in OVCAc. (**A**) Histogram of colony formation in OVCAc treated with sEV from F-OVCAc for 6 days using crystal violet assay; (**B**) Histogram of cell viability using MTT assay in OVCAc treated with sEV from F-OVCAc with/without ferroptotic inhibitors (Ferrostatin-1 (Fer-1) and Deferoxamine (DFO)) for 6 days in OVCA1; (**C**) Histogram of Malondialdehyde (MDA) levels in OVCAc treated with sEV from F-OVCAc with/without ferroptotic inhibitors (Fer-1 and DFO) for 6 days in OVCA1; (**D**) Histogram of Iron (II) (Fe^2+^) levels in OVCAc treated with sEV from F-OVCAc with/without ferroptotic inhibitors (Fer-1 and DFO) for 6 days in OVCA1; (**E**) Histogram of reactive oxygen species (ROS) levels in OVCAc treated with sEV from F-OVCAc with/without ferroptotic inhibitors (Fer-1 and DFO) for 6 days in OVCA1. The graphs show the mean ± SD of three independent experiments. * *p* < 0.05 and ** *p* < 0.01.

**Figure A4:**
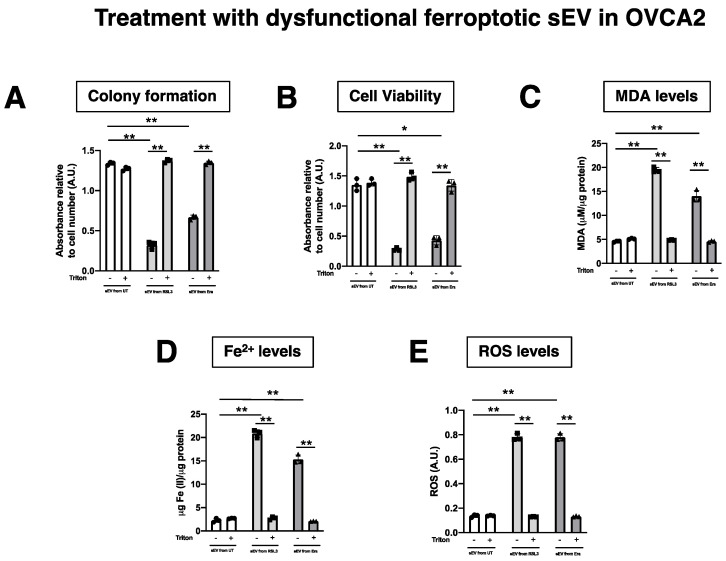
Determination of ferroptosis paracrine transmission using defunctionalized sEV using Triton-X. (**A**) Histogram of colony formation in OVCAc treated with sEV from F-OVCAc for 6 days using crystal violet assay; (**B**) Histogram of cell viability using MTT assay in OVCAc treated with sEV without/ with pre-treatment with Triton-X from F-OVCAc for 6 days in OVCA2; (**C**) Histogram of Malondialdehyde (MDA) levels in OVCAc treated with sEV without/with pre-treatment with Triton-X from F-OVCAc for 6 days in OVCA2; (**D**) Histogram of Iron (II) (Fe^2+^) levels in OVCAc treated with sEV without/with pre-treatment with Triton-X from F-OVCAc for 6 days in OVCA2; (**E**) Histogram of reactive oxygen species (ROS) levels in OVCAc treated sEV without/with pre-treatment with Triton-X from F-OVCAc for 6 days in OVCA2. The graphs show the mean ± SD of three independent experiments. * *p* < 0.05 and ** *p* < 0.01. Related to Figure 8.

To validate the results obtained about ferroptosis transmission by sEV in OVCA, two pharmacological inhibitors of ferroptosis (Ferrostatin (Fer-1) and Deferoxamine (DFO)) were tested. Fer-1 is a ferroptosis inhibitor due to its ability to slow the accumulation of lipid hydroperoxides [17]. DFO is a potent iron chelator, used to treat diseases with iron overload such as traumatic spinal cord injury and myelodysplastic syndromes [18]. Simultaneously with sEV treatment, the drugs were included for six days at the same time as F-OVCA-derived sEV to inhibit the ferroptosis activation (Figure 9). Then, we compared the colony formation, cell viability, MDA, intracellular Fe^2+^ and ROS levels in cells undergoing ferroptosis. As can be seen in Figure 10, sEV isolated from RSL3 and Era induced cell cycle arrest, cell death, and increased lipid peroxidation products (MDA), intracellular Fe^2+^ and ROS (Figure 6 and Figure 8). However, OVCA treated with ferroptotic inhibitors (Fer-1 and DFO) prevented the proliferation arrest, cellular death, and the activation of ferroptosis process with the reduction of MDA, Fe^2+^ and ROS levels (Figure 10 and Figure A4). Altogether, these data validate the ferroptosis transmission mediated by sEV through the accumulation of lipid peroxidation products and intracellular Fe^2+^ in concordance with the data obtained with the dysfunctionality of sEV using Triton-X (Figure 7 and Figure A3).

**Figure 11:**
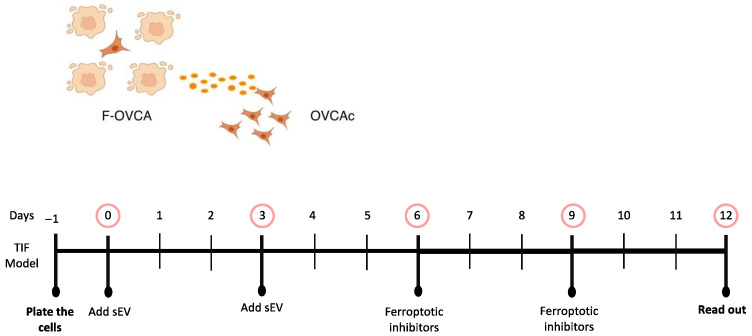
Schematic representation of ferroptotic paracrine through sEV inhibition after starting the process.

**Figure 12:**
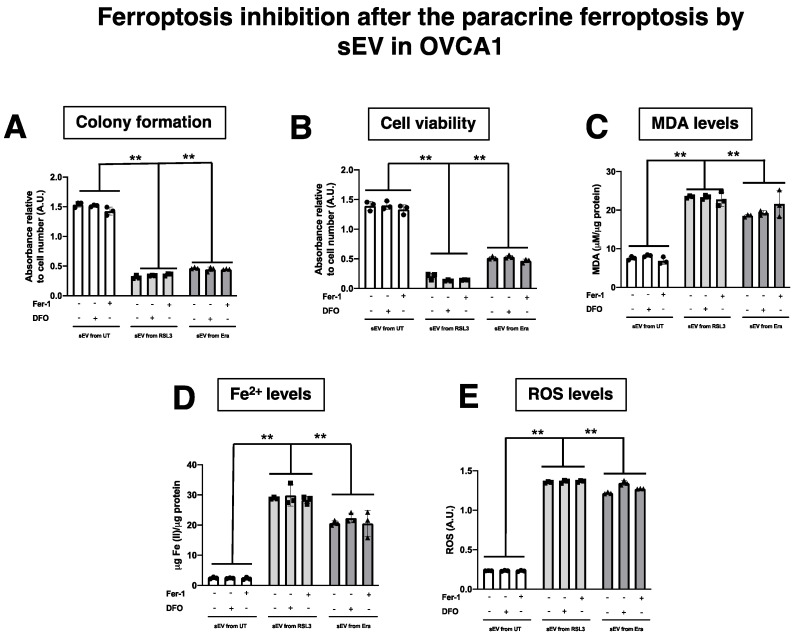
Determination inhibition after the ferroptosis paracrine transmission by sEV in OVCAc. (**A**) Histogram of colony formation using crystal violet assay and (**B**) Histogram of cell viability using MTT assay in OVCAc treated with sEV from F-OVCAc for 6 days and with/without ferroptotic inhibitors (Ferrostatin-1 (Fer-1) and Deferoxamine (DFO)) for 6 days in OVCA1; (**C**) Histogram of Malondialdehyde (MDA) levels in OVCAc treated with sEV from F-OVCAc for 6 days and with/without ferroptotic inhibitors (Fer-1 and DFO) for 6 days in OVCA1; (**D**) Histogram of Iron (II) (Fe^2+^) levels in OVCAc treated with sEV from F-OVCAc for 6 days and with/without ferroptotic inhibitors (Fer-1 and DFO) for 6 days in OVCA1; (**E**) Histogram of reactive oxygen species (ROS) levels in OVCAc treated with sEV from F-OVCAc for 6 days and with/without ferroptotic inhibitors (Fer-1 and DFO) for 6 days in OVCA1. The graphs show the mean ± SD of three independent experiments. ** *p* < 0.01.

**Figure A5:**
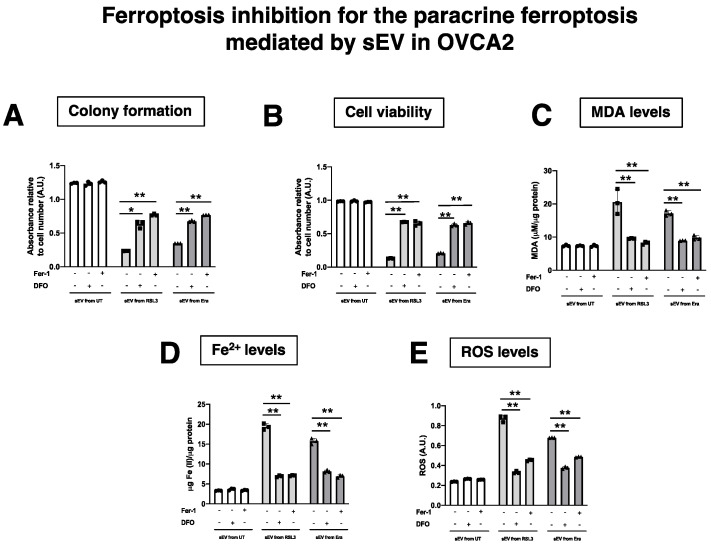
Determination inhibition in the ferroptosis paracrine transmission by sEV in OVCAc. (**A**) Histogram of colony formation in OVCAc treated with sEV from F-OVCAc for 6 days using crystal violet assay; (**B**) Histogram of cell viability using MTT assay in OVCAc treated with sEV from F-OVCAc with/without ferroptotic inhibitors (Ferrostatin-1 (Fer-1) and Deferoxamine (DFO)) for 6 days in OVCA2; (**C**) Histogram of Malondialdehyde (MDA) levels in OVCAc treated with sEV from F-OVCAc with/without ferroptotic inhibitors (Fer-1 and DFO) for 6 days in OVCA2; (**D**) Histogram of Iron (II) (Fe^2+^) levels in OVCAc treated with sEV from F-OVCAc with/without ferroptotic inhibitors (Fer-1 and DFO) for 6 days in OVCA2; (**E**) Histogram of reactive oxygen species (ROS) levels in OVCAc treated with sEV from F-OVCAc with/without ferroptotic inhibitors (Fer-1 and DFO) for 6 days in OVCA2. The graphs show the mean ± SD of three independent experiments. * *p* < 0.05 and ** *p* < 0.01. Related to Figure 10.

Additionally, whether the treatment with DFO and Fer-1 could revert the ferroptotic phenotype induced by sEV (Figure 11) was evaluated. F-OVCA-induced sEV (OVCA1 and OVCA2) were treated with the two inhibitors for six days. Then, the colony formation capacity, cell viability, MDA, Fe^2+^ and ROS levels were evaluated in these cells (Figure 12 and Figure A5). It was determined that the treatment with DFO and Fer-1 does not revert the ferroptotic phenotype in the OVCA cells.

### 3.5. The Effect of sEV from F-OVCA Produced Ferroptosis-Related Biomarkers Is Transmitted

**Figure 13:**
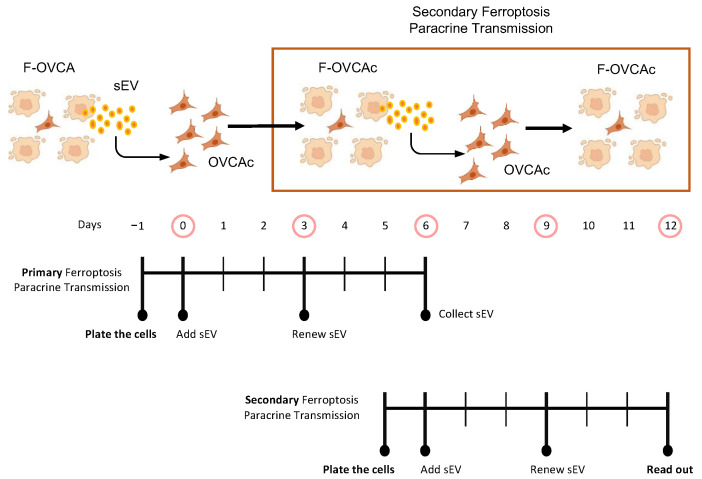
Schematic representation of secondary ferroptotic paracrine through sEV.

**Figure 14:**
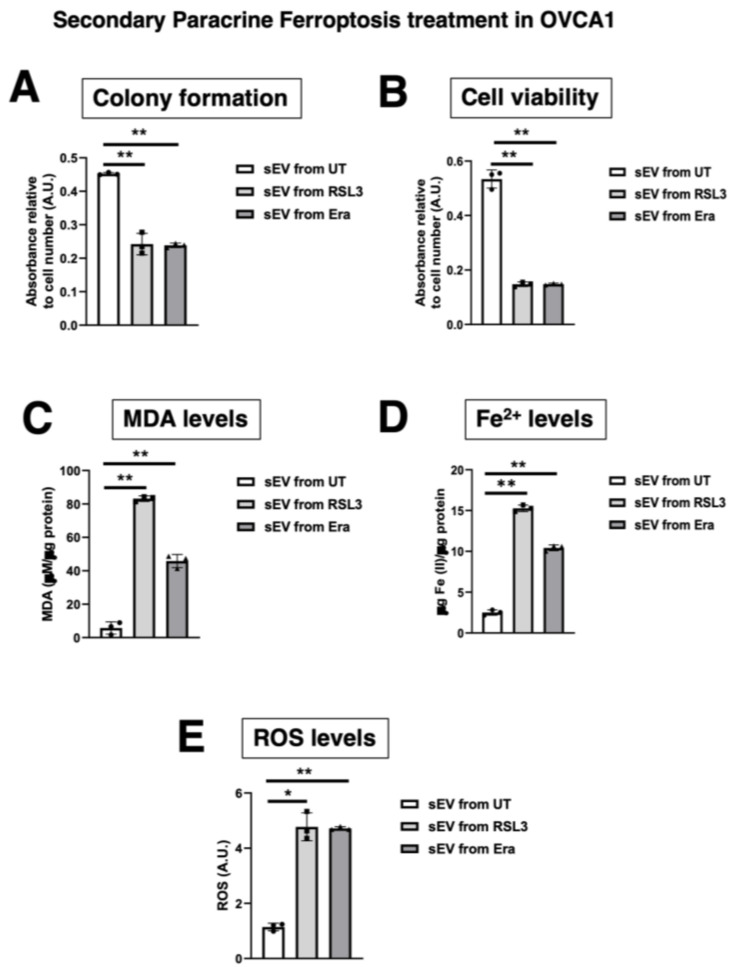
Ferroptosis parameters in secondary ferroptosis paracrine transmission by sEV in OVCAc. (**A**) Histogram of colony formation in OVCAc treated with sEV from OVCAc treated with sEV from F-OVCA-induced by sEV for 6 days in OVCA1; (**B**) Histogram of cell viability using MTT assay in OVCAc treated with sEV from F-OVCAc-induced by sEV for 6 days in OVCA1; (**C**) Histogram of Malondialdehyde (MDA) levels in OVCAc treated with sEV from F-OVCAc-induced by sEV for 6 days in OVCA1; (**D**) Histogram of Iron (II) (Fe^2+^) levels in OVCAc treated with sEV from F-OVCAc- induced with sEV in OVCA1; (**E**) Histogram of reactive oxygen species (ROS) levels in OVCAc treated with sEV from F-OVCA-induced by sEV for 6 days in OVCA1. The graphs show the mean ± SD of three independent experiments. * *p* < 0.05 and ** *p* < 0.01.

**Figure 15:**
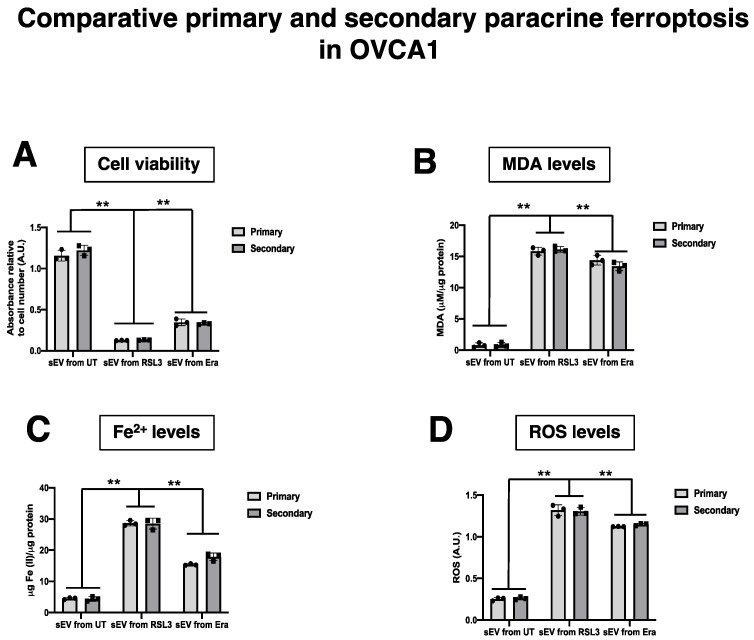
Ferroptosis parameters in the comparative primary and secondary ferroptosis paracrine transmission by sEV in OVCAc. (**A**) Histogram of colony formation in primary and secondary ferroptosis paracrine transmission for 6 days in OVCA1; (**B**) Histogram of cell viability using MTT assay in primary and secondary ferroptosis paracrine transmission for 6 days in OVCA1; (**C**) Histogram of Malondialdehyde (MDA) levels in in primary and secondary ferroptosis paracrine transmission for 6 days in OVCA1; (**C**) Histogram of Iron (II) (Fe^2+^) levels in primary and secondary ferroptosis paracrine transmission for 6 days in OVCA1. (**D**) Histogram of reactive oxygen species (ROS) levels in primary and secondary ferroptosis paracrine transmission for 6 days in OVCA1. The graphs show the mean ± SD of three independent experiments. ** *p* < 0.01.

**Figure A6:**
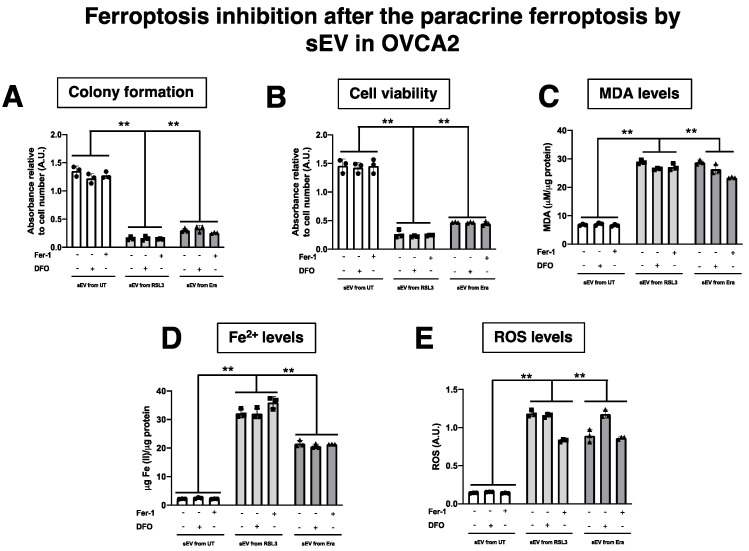
Determination inhibition after the ferroptosis paracrine transmission by sEV in OVCAc. (**A**) Histogram of colony formation using crystal violet assay and (**B**) Histogram of cell viability using MTT assay in OVCAc treated with sEV from F-OVCAc for 6 days and with/without ferroptotic inhibitors (Ferrostatin-1 (Fer-1) and Deferoxamine (DFO)) for 6 days in OVCA2; (**C**) Histogram of Malondialdehyde (MDA) levels in OVCAc treated with sEV from F-OVCAc for 6 days and with/without ferroptotic inhibitors (Fer-1 and DFO) for 6 days in OVCA2; (**D**) Histogram of Iron (II) (Fe^2+^) levels in OVCAc treated with sEV from F-OVCAc for 6 days and with/without ferroptotic inhibitors (Fer-1 and DFO) for 6 days in OVCA2; (**E**) Histogram of reactive oxygen species (ROS) levels in OVCAc treated with sEV from F-OVCAc for 6 days and with/without ferroptotic inhibitors (Fer-1 and DFO) for 6 days in OVCA2. The graphs show the mean ± SD of three independent experiments. ** *p* < 0.01. Related to Figure 12.

Our previous results suggest that sEV can induce ferroptosis in a paracrine manner. Next, we questioned if the F-OVCA generated through sEV treatment could induce a ferroptosis message to OVCA cells. To address this question, sEV were collected from OVCA cells treated with sEV from UT, RSL3 and Era (called primary ferroptosis paracrine). OVCA were treated with sEV from primary ferroptosis paracrine transmission for six days to see if we could induce a secondary ferroptosis paracrine transmission (Figure 13). The capacity to induce ferroptosis in the recipient cells was assessed as previously described. The secondary ferroptosis paracrine transmission was established by a loss of proliferation capacity (Figure 14A), increased cell death (Figure 14B), and increased MDA (Figure 14C), Fe^2+^ (Figure 14D), and ROS levels (Figure 14E). Further, a comparative between primary and secondary ferroptosis paracrine in OVCA cells by the evaluation of ferroptotic parameters (cell viability, MDA, Fe^2+^ and ROS levels) was developed. The data show that there are not statistically significant differences between them in the two OVCA cell lines (Figure 15 and Figure A6).

**Figure 16:**
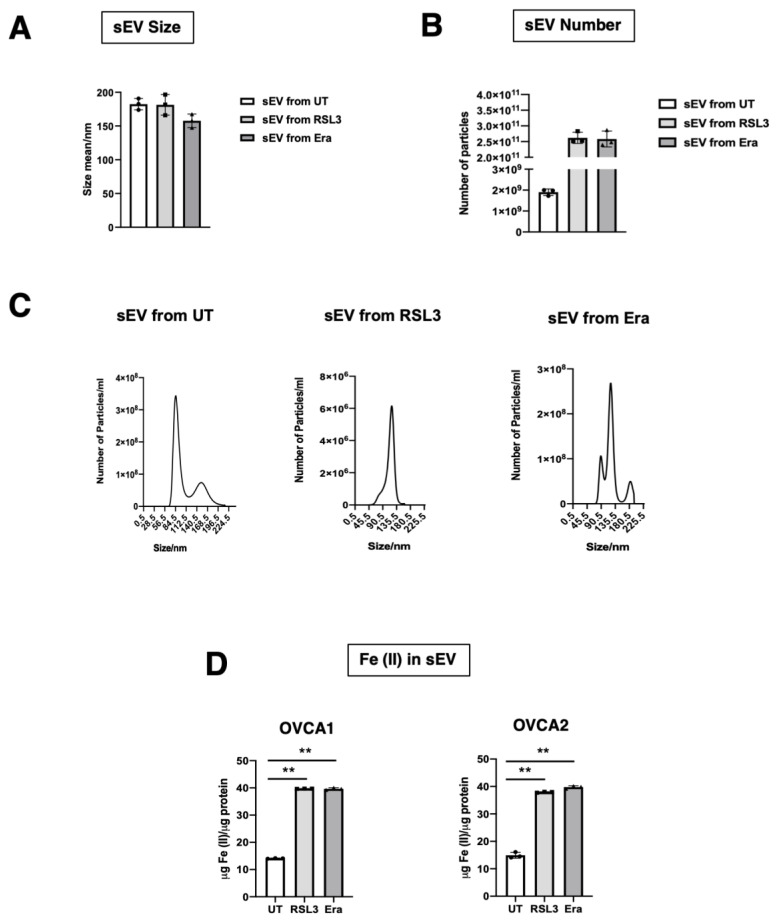
Analysis of sEV delivery from secondary ferroptosis paracrine transmission by sEV in OVCAc. (**A**) Mean particle size and (**B**) number of sEV from F-OVCAc induced by sEV measured by Nanoparticle Tracking Analysis (NTA). (**C**) Histogram distribution of sEV from F-OVCAc induced by sEV by Nanoparticle Tracking Analysis (NTA) in OVCA1 (**D**) Histogram of Iron (II) (Fe^2+^) levels in F-OVCAc induced by sEV in OVCA1 and OVCA2. The graphs show the mean ± SD of three independent experiments. ** *p* < 0.01. Related to Figure 10.

Moreover, the F-OVCA (RSL3 and Era) release a larger number of sEV (Figure 4). In fact, an increase in the sEV production with high contents of Fe^2+^ was observed during secondary ferroptosis paracrine transmission (Figure 16). With these results, it was confirmed that the sEV can induce ferroptosis in OVCA and be further transmitted.

### 3.6. Capacity of Ferroptotic sEV to Activate the Inflammation in Tumor Microenvironment

**Figure 17:**
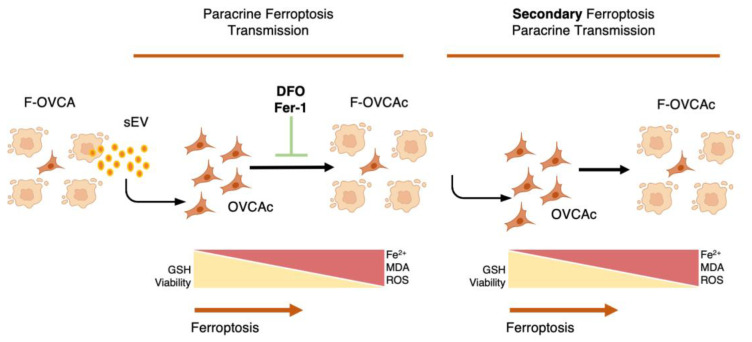
Graphical abstract.

As described throughout this article, ferroptosis is presented as a new process, which provides us with a potential new therapy for epithelial ovarian cancer and improves the quality of patients (Figure 17).

**Figure 18:**
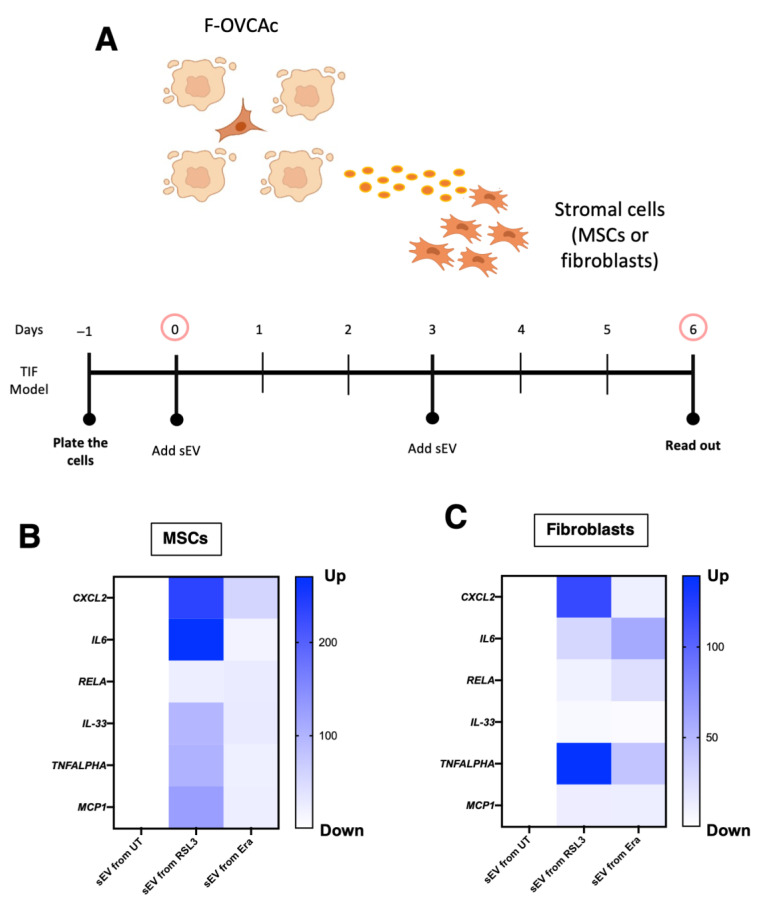
Inflammation activation through ferroptotic sEV in tumor microenvironment. (**A**) Schematic representation of Mesenchymal Stem Cells (MSCs) and fibroblasts treated with ferroptotic sEV. Heatmap of mRNA levels of *CXCL2*, *IL6*, *RELA*, *IL33*, *TNFALPHA* and *MCP1* in (**B**) MSCs and (**C**) fibroblasts treated with sEV from ferroptotic sEV from F-OVCA (OVCA1) for 6 days. Data show the mean of three independent experiments.

The TME is complex and it is implicated in the malignant behavior of tumor cells [19]. In recent years, it was described that ferroptotic cancer cells release signal molecules into the tumor microenvironment to either suppress or promote tumor progression by inflammatory pathways activation [6]. The ferroptotic cells can induce activation of inflammatory pathways such as Toll-like receptor 4 (TLR4) [20]. Because of that, we decided to determine the capacity of ferroptotic sEV in MSCs and fibroblasts, which are important in the TME. MSCs and fibroblasts were treated with sEV from F-OVCA (OVCA1) for six days (Figure 18A). Finally, inflammatory markers (CXCL2, IL6, RELA, IL33, TNFALPHA and MCP1) were checked using qPCR-RT in the recipient MSCs and fibroblasts (Figure 18B,C). The activation of the inflammation phenotype in the stroma cells was determined through F-OVCA-derived sEV.

## 4. Discussion

EOC patients develop drug resistance, high risk of metastasis after the treatment with chemotherapy, angiogenesis inhibitors, and Poly (ADP-ribose) polymerases (PARPs) inhibitors, among others. All of this is associated with a poor prognosis [1]. Moreover, undiscovered new metabolic pathways with pharmacological potential could help to overcome the EOC and increase the survival rate.

On the one hand, the bio element iron has gained importance because it is a regulator of important biological processes such as oxygen transport, DNA biosynthesis and ATP generation [17]. On the other hand, iron is involved in the redox process and increases the accumulation of ROS and the initiation of signaling pathways implicated in survival and cell death [18]. Conversely, in breast and epithelial ovarian cancer cells, it was determined that iron is involved in the tumor initiation, progression, metastasis, and tumor microenvironment [21].

Ferroptosis is a type of regulated cell death distinct from other modalities mechanistically and functionally as apoptosis, autophagy, and necrosis [17], triggered by the accumulation of oxidative damage in the lipid membrane, the accumulation of ROS and free iron. In the last year, it was reported that ferroptosis has an anti-tumor therapeutic potential in breast cancer [9,10], lung cancer [5,22], hepatocellular carcinoma [23] and melanoma, among others [24].

Our data are a preliminary approach for a new therapeutic line of research in the field of ferroptosis and EOC to improve the conventional treatments against EOC. Recently, it was determined that ferroptosis is regulated by glutathione peroxidase 4 (GPX4) and ferroptosis suppressor protein (FSP1) in OVCA cells [4,5]. However, the role of intercellular communication in ferroptosis is poorly studied. Because of that, we studied how the ferroptosis is transmitted in a paracrine manner in EOC.

The intercellular communication in EOC is involved in the promoting of the epithelial–mesenchymal transmission and the TME [25]. The sEV regulate the accumulation of lipid peroxidation product and ROS [11]. Evidences exist of ferroptosis-mediated crosstalk in the TME of hepatocellular carcinoma, pancreatic, breast and lung cancer by activation of the inflammatory pathway in stroma and immune cells [6].

Non-cell autonomous (paracrine) ferroptosis via sEV has been previously described as an important mechanism in ferroptosis resistance in lung [22] and breast cancer [9,10], although these studies do not study the ferroptosis transmission effect of soluble factors and sEV. Here, we provide evidence that sEVs are responsible for mediating paracrine ferroptosis in OVCA cells. Through the use of pharmacological inhibitors of ferroptosis such as DFO and Fer-1, iron chelator and lipophilic radical trap, respectively [22,26], for the sEV treatment, we validated the ferroptosis activation by sEV. However, we cannot discard that DFO and Fer-1 can affect the sEV uptake in the recipient cells. However, we confirmed the ferroptotic transmission message by sEV using dysfunctionality sEV pre-treated with a detergent as Triton-X. With the previous, we discovered that ferroptosis transmission can be further transmitted.

Two “mechanisms” have been described in the literature by which ferroptotic cells could be acting as transmitting agents contributing to the propagation of the process. On the one hand, treatment of non-tumor and tumor mammary epithelial cells with the exogenous modulators of ferroptosis such as RSL3 and Era, increases the expression levels of prominin2. Prominin2 is a pentaspanin that promotes the formation of multivesicular bodies and exosomes which include ferritin, a protein involved in iron storage [9]. The presence of ferritin in exosomes from ferroptotic cells has been observed in other cell types such as macrophages. Ferritin-loaded sEV can be taken up by neighboring cells [3]. On the other hand, ferroptotic murine embryonic fibroblasts (MEFs) obtained after treatment with RSL3 and Era are also capable of transmitting this process by propagating, in this case, lipid peroxidation, probably through lipid mediators [27]. We have not studied whether either of these two mechanisms, together or separately, are working in this case. However, we have evaluated the levels of Fe^2+^ in sEV from two different cell lines, SKVO3 and A2780, treated with the two ferroptotic inducers used and with sEV from ferroptotic cells, and it was observed that Fe^2+^ levels increased after the different treatments. Although further studies are needed to determine the mechanism by which these cells can transmit this process through sEV, we propose that, because Fe^2+^ levels are increased, the transmission could be mediated by ferritin.

Furthermore, the F-OVCA cells are able to generate an amount of sEV contributing to much higher ferroptosis paracrine transmission in an in vivo context besides the activation of inflammation phenotype in stroma cells. This manuscript is a proof-of-concept to determine the ferroptosis transmission by sEV in EOC.

## 5. Conclusions

This study has, for the first time, provided evidence of the capacity of ovarian cancer cells treated with ferroptotic inducers to modify their intercellular environment and communicate with surrounding cells. Thus, it could open up other research avenues of EOC treatment, ferroptotic modulators being a feasible one.

## Figures and Tables

**Table 1:** List of ferroptotic modulators.

Drug	Concentration	Source/Cat No
RSL3	250 nM, 500 nM and 1 µM	MedChemExpress/HY-100218A
Erastin	250 nM, 500 nM and 1 µM	MedChemExpres/HY-15763
Ferrostatin-1	500 nM	MedChemExpres/HY-100579
DFO	500 nM	MedChemExpres/HY-B0988

**Table 2:** List of antibodies.

Antibody	Dilution	Source/Cat No
CD63	1:5000	Abcam/ab231975
Calnexin	1:1000	Abcam/ab133615

**Table 3:** List of primers used in qPCR-RT.

Target	Species	Forward Primer (5′-3′)	Reverse Primer (3′-5′)
*CXCL2*	Human	CTCAAGAATGGGCAGAAAGC	AAACACATTAGGCGCAATCC
*IL6*	Human	CCAGGAGCCCAGCTATGAAC	CCCAGGGAGAAGGCAACTG
*RELA*	Human	TTCCCGATCTGAGTCCAGGT	GCTTGTCTCGGGTTTCTGGA
*IL33*	Human	TCCTTGCTTGGCAGTATCCA	TGCTCAATGTGTCAACAGACG
*TNFALPHA*	Human	AGCCCATGTTGTAGCAAACC	GAGGTACAGGCCCTCTGATG
*MCP1*	Human	TCAGCCAGATGCAATCAATG	ATGGTCTTGAAGATCACAGC
*ACTB*	Human	AGAGCTACGAGCTGCCTGAC	GGATGCCACAGGACTCCA

**Figure A7:**
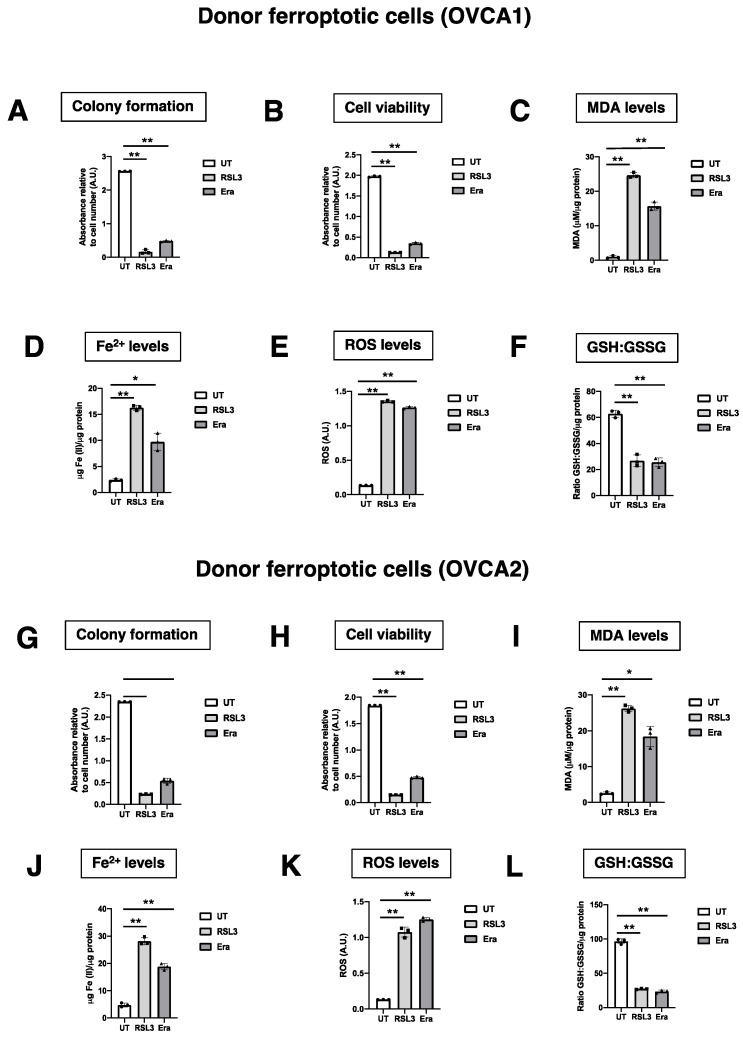
Ferroptosis parameters in the donor ferroptotic cells for comparative primary and secondary ferroptosis paracrine transmission by sEV in OVCAc. (**A**) Histogram of colony formation donor ferroptotic OVCA1; (**B**) Histogram of cell viability using MTT assay in donor ferroptotic OVCA1; (**C**) Histogram of Malondialdehyde (MDA) levels in donor ferroptotic OVCA1; (**D**) Histogram of Iron (II) (Fe^2+^) levels in donor ferroptotic OVCA1; (**E**) Histogram of reactive oxygen species (ROS) levels in donor ferroptotic OVCA1. (**F**) Histogram of GSH:GSSG levels in donor ferroptotic OVCA1; (**G**) Histogram of colony formation donor ferroptotic OVCA2; (**H**) Histogram of cell viability using MTT assay in donor ferroptotic OVCA2; (**I**) Histogram of Malondialdehyde (MDA) levels in donor ferroptotic OVCA2; (**J**) Histogram of Iron (II) (Fe^2+^) levels in donor ferroptotic OVCA2; (**K**) Histogram of reactive oxygen species (ROS) levels in donor ferroptotic OVCA2. (**L**) Histogram of GSH:GSSG levels in donor ferroptotic OVCA2. The graphs show the mean ± SD of three independent experiments. * *p* < 0.05 and ** *p* < 0.01. Related to Figure 15.

## Data Availability

Not applicable.
